# Proteomes reveal metabolic capabilities of *Yarrowia lipolytica* for biological upcycling of polyethylene into high-value chemicals

**DOI:** 10.1128/msystems.00741-23

**Published:** 2023-10-26

**Authors:** Caleb Walker, Max Mortensen, Bindica Poudel, Christopher Cotter, Ryan Myers, Ikenna O. Okekeogbu, Seunghyun Ryu, Bamin Khomami, Richard J. Giannone, Siris Laursen, Cong T. Trinh

**Affiliations:** 1 Department of Chemical and Biomolecular Engineering, University of Tennessee, Knoxville, Tennessee, USA; 2 Biosciences Division, Oak Ridge National Laboratory, Oak Ridge, Tennessee, USA; University of California, Davis, California, USA

**Keywords:** plastic waste, polyethylene, LLDPE, upcycling, *Yarrowia lipolytica*, catalytic depolymerization, n-alkanes, 1-alkenes, citric acid, n-hexadecane, neutral lipids, adaptive laboratory evolution, proteome, proteome reallocation

## Abstract

Polyolefins derived from plastic wastes are recalcitrant for biological upcycling. However, chemical depolymerization of polyolefins can generate depolymerized plastic (DP) oil, comprising of a complex mixture of saturated, unsaturated, even, and odd hydrocarbons suitable for biological conversion. While DP oil contains a rich carbon and energy source, it is inhibitory to cells. Understanding and harnessing robust metabolic capabilities of microorganisms to upcycle the hydrocarbons in DP oil, both naturally and unnaturally occurring, into high-value chemicals are limited. Here, we discovered that an oleaginous yeast, *Yarrowia lipolytica,* undergoing short-term adaptation to DP oil robustly utilized a wide range of hydrocarbons for cell growth and production of citric acid and neutral lipids. When growing on hydrocarbons, *Y. lipolytica* partitioned into planktonic and oil-bound cells with each exhibiting distinct proteomes and amino acid distributions invested in establishing these proteomes. Significant proteome reallocation toward energy and lipid metabolism, belonging to 2 of the 23 Eukaryotic Orthologous Groups classes C and I, enabled robust growth of *Y. lipolytica* on hydrocarbons, with n-hexadecane as the preferential substrate. This investment was even higher for growth on DP oil where classes C and I were ranked one and two, respectively, and many associated proteins and pathways were expressed and upregulated including the hydrocarbon degradation pathway, Krebs cycle, glyoxylate shunt and, unexpectedly, propionate metabolism. However, a reduction in proteome allocation for protein biosynthesis, at the expense of the observed increase toward energy and lipid metabolisms, might have caused the inhibitory effect of DP oil on cell growth.

**IMPORTANCE:**

Sustainable processes for biological upcycling of plastic wastes in a circular bioeconomy are needed to promote decarbonization and reduce environmental pollution due to increased plastic consumption, incineration, and landfill storage. Strain characterization and proteomic analysis revealed the robust metabolic capabilities of *Yarrowia lipolytica* to upcycle polyethylene into high-value chemicals. Significant proteome reallocation toward energy and lipid metabolisms was required for robust growth on hydrocarbons with n-hexadecane as the preferential substrate. However, an apparent over-investment in these same categories to utilize complex depolymerized plastic (DP) oil came at the expense of protein biosynthesis, limiting cell growth. Taken together, this study elucidates how *Y. lipolytica* activates its metabolism to utilize DP oil and establishes *Y. lipolytica* as a promising host for the upcycling of plastic wastes.

## INTRODUCTION

Plastics are hydrocarbons that are mainly derived from petroleum (1). Its high demand and production with limited recyclability generate plastic wastes that have been polluting our ecosystems for decades, negatively impacting both wildlife and human health (1, 2). Yet, sustainable processes to recycle plastic wastes remain elusive since current recycling technologies produce polymers with lower quality and higher cost (3). One solution to improve the sustainability and cost competitiveness of the recycling process is to upcycle plastic wastes into high-value chemicals (4). Due to the abundance of hydrocarbons in plastic wastes such as polyolefins (e.g., polyethylene and polypropylene), efficient biological upcycling of these wastes into high-value chemicals at standard conditions, such as room temperature and atmospheric pressure, is both disruptive and transformative (5).

In nature, certain microorganisms have been discovered for their unique metabolic capability to degrade plastic (6, 7). However, direct microbial depolymerization of plastic wastes, especially those derived from complex and recalcitrant polyolefins, is inefficient due to slow degradation rates that take years to complete (7–9). One strategy to overcome this limitation is to first depolymerize plastic wastes in a thermochemical pretreatment step to generate low-molecular weight depolymerized plastic (DP) intermediates (e.g., C11–C28 hydrocarbons in a form of oil and wax) (10) that microorganisms can rapidly and effectively utilize for biosynthesis of high-value chemicals with high selectivity and ease of separation in the biological upcycling step. This strategy resembles an integrated biorefinery for conversion of lignocellulosic biomass to biofuels and biobased products where chemical pretreatment is first performed to reduce biomass recalcitrance followed by saccharification and fermentation to produce target products (11).

For thermochemical pretreatment, catalytic cleavage of polyolefins to C11–C28 hydrocarbon intermediates that microorganisms can utilize is more advantageous than less controlled methods such as non-catalytic pyrolysis or direct functionalization (12–15). The catalytic approach enables the reaction to be accelerated at lower temperatures than pyrolysis and can provide far greater control over product distributions (16–18). As polyolefins are large, saturated hydrocarbons, solid acid catalysts (e.g., SiC or its derivatives) are commonly employed due to the well-known surface chemistry of acidic sites in hydrocarbon activation, cleavage, and isomerization (19–24). The elementary mechanism for solid acid-catalyzed saturated hydrocarbon cleavage proceeds through dehydrogenation, C-C bond cleavage, and either desorption or re-hydrogenation to produce unsaturated or saturated products in a range of C11–C28 hydrocarbon intermediates suitable for biological conversion (19, 20, 25). Depending on reaction temperature, hydrogenation kinetics over solid acid catalysts can be kinetically slow; thus, metal co-catalysts might need to be employed to improve the saturation of products (26–29). For biological upcycling, the resulting hydrocarbon intermediates contain rich carbon and electron sources that enable microorganisms to grow and produce high-value chemicals. However, mixed and complex hydrocarbons with saturated, unsaturated, linear, branched, even, and/or odd carbon chains can be inhibitory and non-degradable to microorganisms since they have not yet adapted to utilize these hydrocarbons. Therefore, biological upcycling of depolymerized plastics to produce high-value chemicals requires efficient and robust microorganisms.

One promising microbial host that can be repurposed for biological upcycling of hydrocarbon intermediates is the generally-regarded-as-safe (GRAS certified) oleaginous yeast *Yarrowia lipolytica* (30, 31). This organism has broad industrial use with the unique metabolic capability to efficiently produce high-value chemicals (e.g., organic acids [32–35], erythritol [36], and lipid-derived products [37–41]), has tractable genetics with facile genetic manipulation (42), and demonstrates natural robustness, being able to thrive in a broad range of pH (43), high salinity (44), organic solvents such as ionic liquids (45–47), and inhibitory biomass hydrolysates (48, 49). Since *Y. lipolytica* was isolated from fat-rich environments such as cheese and sausage (50), it is also known to naturally assimilate even and saturated C10–C18 n-alkanes (or paraffins), with n-hexadecane (C16) as the most preferable substrate (31, 51, 52).

The n-alkane degradation process is complex. *Y. lipolytica* first secretes an emulsifier (e.g., liposan) to sequester alkanes for targeted uptake through cell adhesion and cellular protrusions (53–55). Intracellular n-alkane degradation involves multiple enzymatic steps across several subcellular compartments including the cytosol, endoplasmic reticulum (ER), peroxisome, and mitochondria (56). In the ER, n-alkanes are oxidized to fatty alcohols by cytochrome P450s (57, 58), then to fatty aldehydes by fatty alcohol dehydrogenases and/or fatty alcohol oxidases (59), and finally to fatty acids by fatty aldehyde dehydrogenases (60). Once transported into the peroxisome, fatty acids are converted to fatty acyl-CoAs by ligases and degraded through the beta-oxidation cycle (56, 61). The terminal metabolite of the n-alkane degradation in the peroxisome is acetyl-CoA, a building block for cell growth and product synthesis. In mitochondria, acetyl-CoA enters the Krebs cycle, generating energy and producing dicarboxylic acids (e.g., citric acid, alpha-ketoglutaric acid, and succinic acid). However, in the cytosol, it is directed toward lipid biosynthesis. Even though some metabolic processes involved in *Y. lipolytica*’ s n-alkane degradation have been characterized, it is still largely unknown how *Y. lipolytica* utilizes DP intermediates derived from plastic wastes (e.g., polyolefins) that contain a complex mixture of hydrocarbons including odd and/or unsaturated alkenes.

In this study, we generated depolymerized polyethylene by thermocatalytic pretreatment and investigated the robustness of *Y. lipolytica* for biological upcycling of the mixed and complex (C11–C28) hydrocarbons. Through a short-term adaptation, we isolated an adapted *Y. lipolytica* and characterized its ability to utilize the inhibitory DP intermediates for growth and production of citric acid and lipids. We elucidated the robust cellular processes of *Y. lipolytica* for utilizing these complex hydrocarbons and quantitatively determined how *Y. lipolytica* allocated its proteome for cell growth and product formation.

## RESULTS

### Catalytic depolymerization of low linear density polyethylene with SiC catalyst generated DP intermediates suitable for microbial conversion

**Fig 1:**
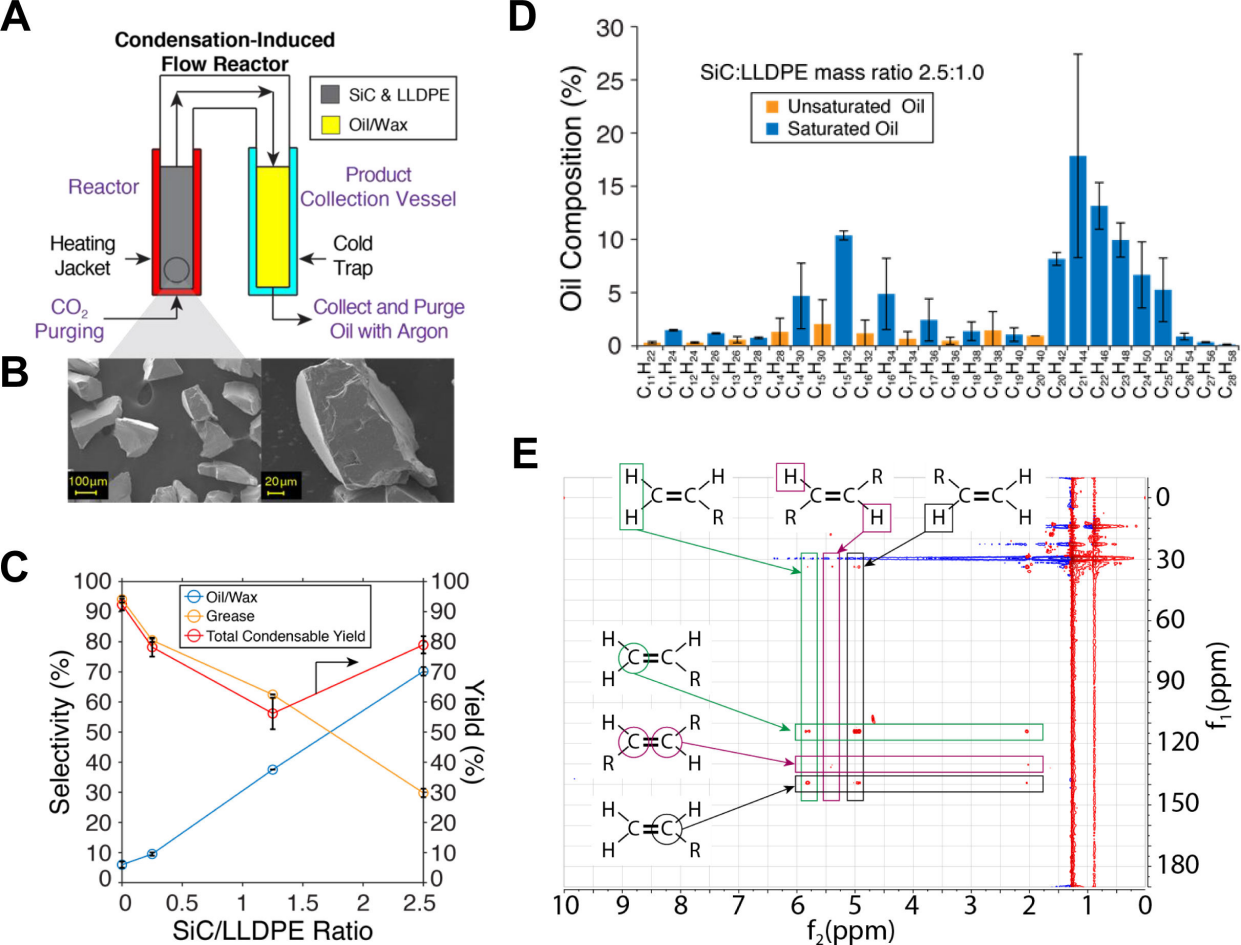
(**A**) Scheme of a condensation-induced flow reactor built for thermocatalytic depolymerization of plastics. (**B**) SEM micrograph of the SiC catalyst. (**C**) Selectivities and yields for LLDPE depolymerization in a semi-batch reactor with a range of SiC-to-LLDPE ratios. Reactions were performed at 400°C for 8 hours under an inert gas of CO_2_. (**D**) Composition of DP oil/wax analyzed by gas chromatography coupled with mass spectrometer (GCMS). (**E**) Analysis of (un)saturation of DP oil/wax by 1H-13C HMBC nuclear magnetic resonance (NMR).

To generate depolymerized plastic intermediates that are compatible with biological upcycling by *Y. lipolytica*, we targeted the production of saturated, linear C11–C18 hydrocarbons as DP oil. As a proof-of-study, we investigated catalytic depolymerization of low linear density polyethylene (LLDPE) as a model polyolefin waste in a static bed reactor with a condensation flow under an inert gas of CO_2_ (Fig. 1A) using an unmodified SiC catalyst (Fig. 1B). The surface composition of SiC is a suboxide of SiO, which presents weak acid sites to drive polyolefin cleavage. Since the polymer itself autogenously provides atomic hydrogens for the hydrogenation of surface-bound cleavage reaction intermediates, no external hydrogen source was added. A static reaction bed was dewatered, and the reaction proceeded at 400°C for 8 hours. To maximize favorable oil production, we manipulated the catalyst to polymer ratios of 0, 0.25, 1.25, and 2.5.

Thermocatalytic depolymerization of LLDPE generated four types of product streams, including grease, oil, wax, and low-molecular weight hydrocarbon off-gases. The reaction without the SiC catalyst resulted in an overall condensable yield of 91% with a grease selectivity of 95% and an oil/wax selectivity of 5% (Fig. 1C). Introduction of SiC at a SiC:LLDPE ratio of 0.25 produced a higher oil/wax selectivity of 18% but the total condensable yield decreased to 81%. With a SiC:LLDPE ratio of 1.25, the oil/wax selectivity increased to 41% with a drastic decrease in total condensable yield down to 55%. With a SiC:LLDPE ratio of 2.5, the oil/wax selectivity further improved to 72% and the total condensable yield increased to 78%. These results illustrate the role of thermal radicals in the formation of the grease product at 400°C when no catalyst was present. Access to the catalytic surface chemistry of SiC was demonstrated by a monotonic increase of wax and oil yield as catalyst to polymer ratio increased. Unlike the oil/wax yields, the total condensable yields did not track monotonically with the catalyst-to-polymer ratios, which underlines the significant role of mass transport limitation in the reaction.

Through the GCMS and 1H-13C HMBC NMR analyses, we found that the oil/wax product consisted of mostly saturated linear hydrocarbon chains of size C_11_–C_28_ and exhibited a bi-modal product distribution with modes centered at C_15_ and C_21_ (Fig. 1D and E). Estimated saturation by GCMS was found to be 96% with only 4% unsaturated hydrocarbons. Characterization of the oil by 1H–13C HMBC NMR (Fig. 1E) indicated that end-chain unsaturation dominated the distribution of unsaturated products. Gel permeation chromatography of the grease product indicated an average molecular weight of 69,630 g/mol.

Overall, the thermocatalytic depolymerization of LLDPE in a condensation-induced flow reactor using SiC catalyst with SiC:LLDPE of 2.5:1.0 generated good yields and selectivities of oil/wax product streams that can be suitable for downstream bioprocessing. The next step was to characterize and elucidate the compatibility of *Y. lipolytica* for biological upcycling of DP oil.

### *Y. lipolytica* used DP oil as carbon and energy sources for growth

#### *Y. lipolytica* was capable of growing on DP oil

**Fig 2:**
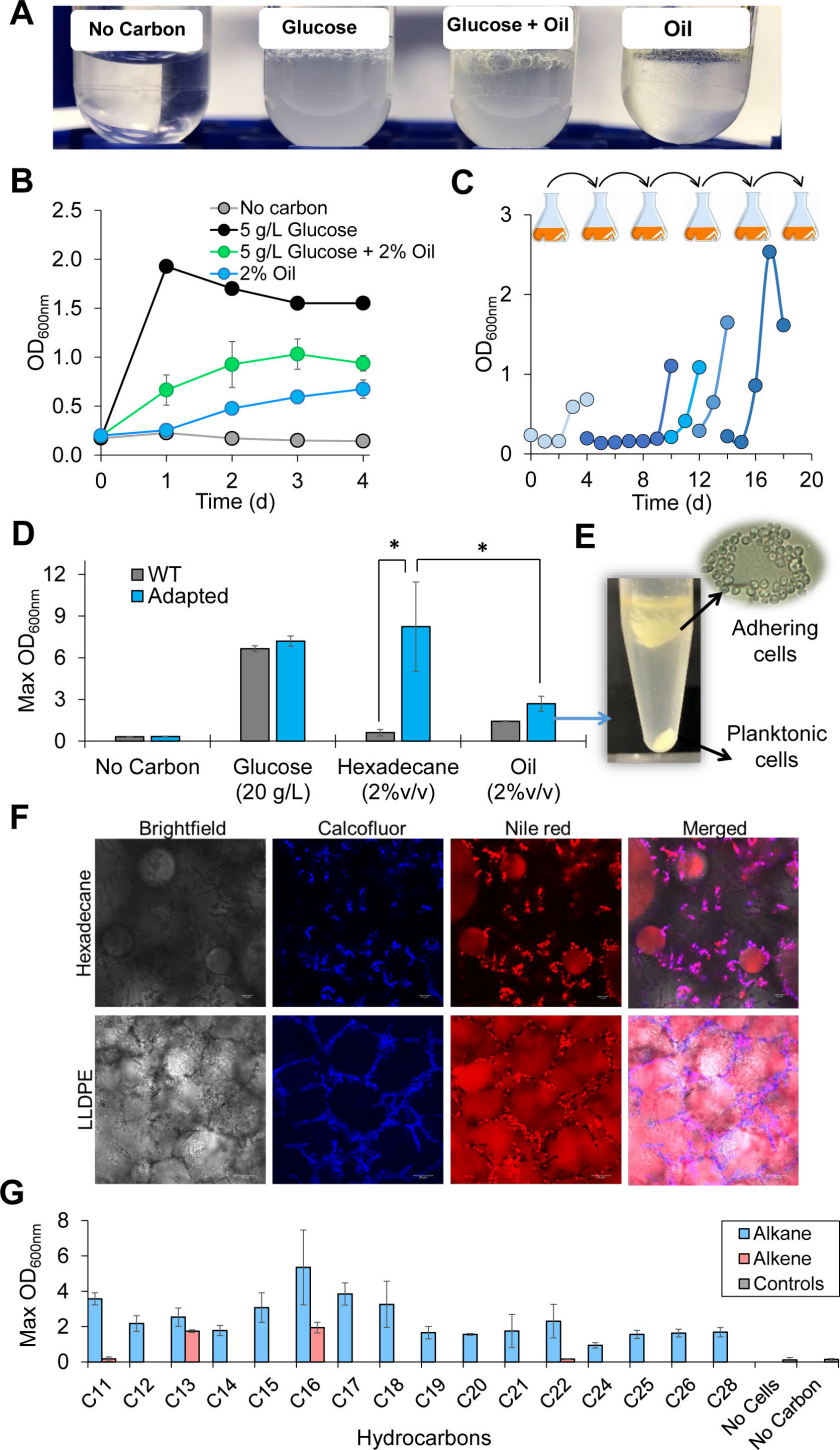
Comprehensive growth characterization of *Y. lipolytica* in hydrocarbons. (**A, B**) Growth of the parent *Y. lipolytica* Po1f in (**A**) culture tubes and (**B**) baffled flasks. (**C**) Short-term adaptation of *Y. lipolytica* in DP oil to generate adapted strain. (**D**) Growth comparison of the parent and adapted *Y. lipolytica* in glucose, n-hexadecane, and DP oil. Symbol: *P < 0.001 using one-way analysis of variance Holm-Sidak method. (**E**) Image of adapted *Y. lipolytica* cultured in DP oil after centrifugation and microscopic image of adhering (or bound) cells. (**F**) Confocal imaging of oil-bound *Y. lipolytica* cells growing on n-hexadecane and DP oil. Calcofluor white stains the chitin cell walls while nile red stains the lipid bodies inside the cells and the oil droplets outside the cells. (**G**) Growth of DP-adapted *Y. lipolytica* on individual n-alkanes (blue) and 1-alkenes (red).

Initial characterization of the parent *Y. lipolytica* Po1f showed cell growth (i.e., turbidity) in culture tubes containing 5% (vol/vol) DP oil as a sole carbon source and in culture tubes containing a mixture of 5% (vol/vol) DP oil and 5 g/L glucose after 2 days of cultivation (Fig. 2A). To confirm cell growth on DP oil, we repeated this experiment using baffled flasks to provide better aeration for cell growth. We found that 2% (vol/vol) DP oil as a sole carbon source supported growth of *Y. lipolytica* cells (Fig. 2B). However, cell growth was not detected using 5% or 10% (vol/vol) DP oil. Notably, maximum cell density (i.e., optical density at 600 nm absorbance [OD_600_]) in a mixture of glucose and DP oil was reduced by ~53% in comparison to cell growth in glucose alone (Fig. 2B). These results indicate DP oil is inhibitory to *Y. lipolytica*.

#### Short-term adaptation of *Y. lipolytica* significantly enhanced cell growth on DP oil

To improve cell growth on DP oil, we employed a short-term adaptation experiment by subjecting wild-type *Y. lipolytica* to five successive transfers in 2% (vol/vol) oil (Fig. 2C). Short-term adaptation (~10 generations) improved growth performance in DP oil, enabling the adapted strain (maximum OD_600_ of 2.69 ± 0.55) to increase cell density ~1.9-fold as compared with the parent strain (maximum OD_600_ of 1.43 ± 0.04) using 2% (vol/vol) DP oil as a sole carbon source (Fig. 2D). Compared with DP oil, the adapted strain demonstrated superior growth (maximum OD_600_ of 8.24 ± 0.55) on the model substrate n-hexadecane, a component of DP oil, by 3.1-fold (Fig. 2D). In addition to observing planktonic cells in the cultures, we found that the adapted strain exhibited cellular adhesion to the hydrophobic layer of both DP oil and n-hexadecane even after centrifugation (Fig. 2E and F).

### Adapted *Y. lipolytica* exhibited improved growth on a broad range of saturated n-alkanes and some select 1-alkenes present in DP oil/wax

While *Y. lipolytica* is known to grow on linear, even, and saturated C10–C18 paraffins (oil form) (31, 51, 52), it has not yet been shown to utilize longer n-alkanes (wax form), 1-alkenes, or odd-chain hydrocarbons that are also present in DP intermediates. To better understand how *Y. lipolytica* could utilize these types of DP intermediates (oil/wax), we next characterized and compared cell growth of adapted *Y. lipolytica* across a variety of individual hydrocarbons. Interestingly, all (C11–C28) odd and even n-alkanes supported growth of DP oil-adapted *Y. lipolytica* as the sole carbon sources (Fig. S1). Notably, cell growth on (C16) n-hexadecane outperformed all other hydrocarbons tested (Fig. 2G). However, among a smaller library of 1-alkenes tested, only medium carbon chain length 1-alkenes (i.e., 1-C13 and 1-C16) supported cell growth, whereas cells failed to grow with short (i.e., 1-C11) or long (i.e., 1-C22) 1-alkenes as the sole carbon sources (Fig. 2G and 1). Taken together, *Y. lipolytica* has the metabolic capability to utilize the complex mixture of alkanes and alkenes in the DP oil derived from polyethylene. Medium-chain saturated hydrocarbons (e.g., C11–C18) best supported the growth of *Y. lipolytica*.

### *Y. lipolytica* upcycled DP oil to citric acid and lipids

**Fig 3:**
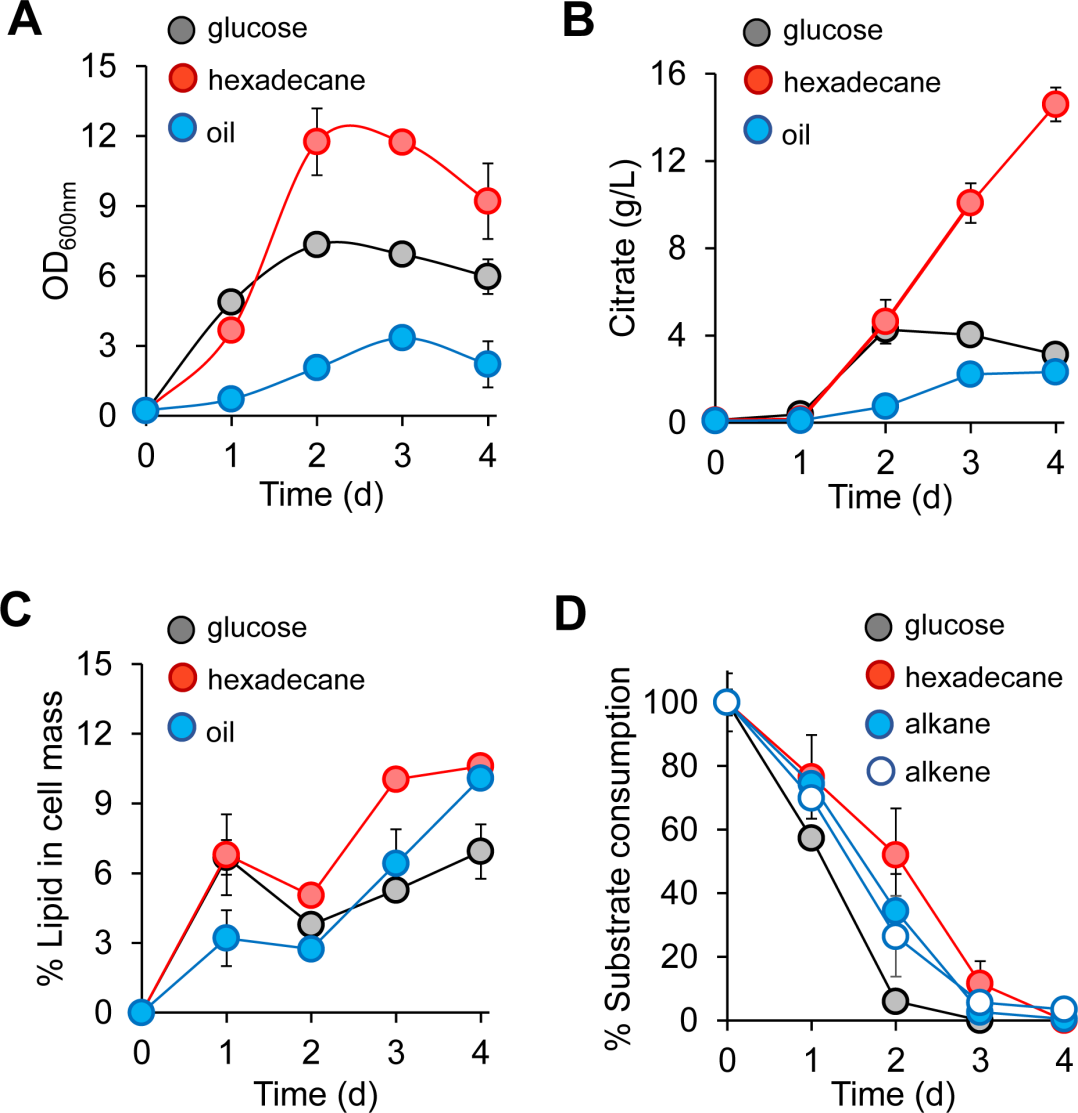
Biological upcycling of DP oil by adapted *Y. lipolytica* for production of citric acid and neutral lipids. (**A**) Growth kinetics of adapted *Y. lipolytica* on DP oil and control substrates (glucose and n-hexadecane), (**B**) citric acid production, (**C**) lipid accumulation, and (**D**) substrate consumption profiles.

Next, we investigated whether *Y. lipolytica* could upcycle DP oil into higher-value chemicals, specifically triacylglycerol lipids and citric acid, using nitrogen limited (C:N = 100) media. We also used glucose and n-hexadecane as controls. Using 2% (vol/vol) DP oil, adapted *Y. lipolytica* achieved a maximum cell density of 3.34 ± 0.32 OD_600_ (Fig. 3A) while producing 2.33 ± 0.12 g/L citric acid (Fig. 3B) and accumulating 10.09 ± 0.42% lipid in cell mass (Fig. 3C). However, maximum cell density (11.74 ± 0.36 OD_600_) and citric acid production (14.6 ± 0.77 g/L citric acid) were significantly higher in 2% (vol/vol) n-hexadecane than in the DP oil (Fig. 3A and B). Both n-hexadecane and DP oil carbon sources exhibited similar lipid levels by day 4 (Fig. 3C). Cells grown in 20 g/L glucose showed the lowest lipid level (6.93 ± 1.17% lipid in cell mass) with moderate citric acid titer (4.28 ± 0.37 g/L citric acid) and intermediate cell growth (7.34 ± 0.14 OD_600_) between the three carbon sources (Fig. 3A and C). Interestingly, both n-hexadecane and DP oil GCMS profiles showed complete consumption (Fig. 3D) despite the differences in cell growth and citric acid production. Cultivation with only n-hexadecane enabled superior growth of *Y. lipolytica* and its production of citric acids and lipids, especially relative to the DP oil. While adapted *Y. lipolytica* could consume DP oil, its growth was limited because the mixed and complex composition of the oil might have limited cellular metabolism impacting substrate assimilation and hence growth and product formation. To understand these differences, we next employed proteomics to elucidate how *Y. lipolytica* utilized hydrophobic hydrocarbons.

### Proteomes revealed the robust metabolic capability of *Y. lipolytica* for utilizing n-hexadecane

#### Planktonic and bound cells exhibited distinct proteomes

**Fig 4:**
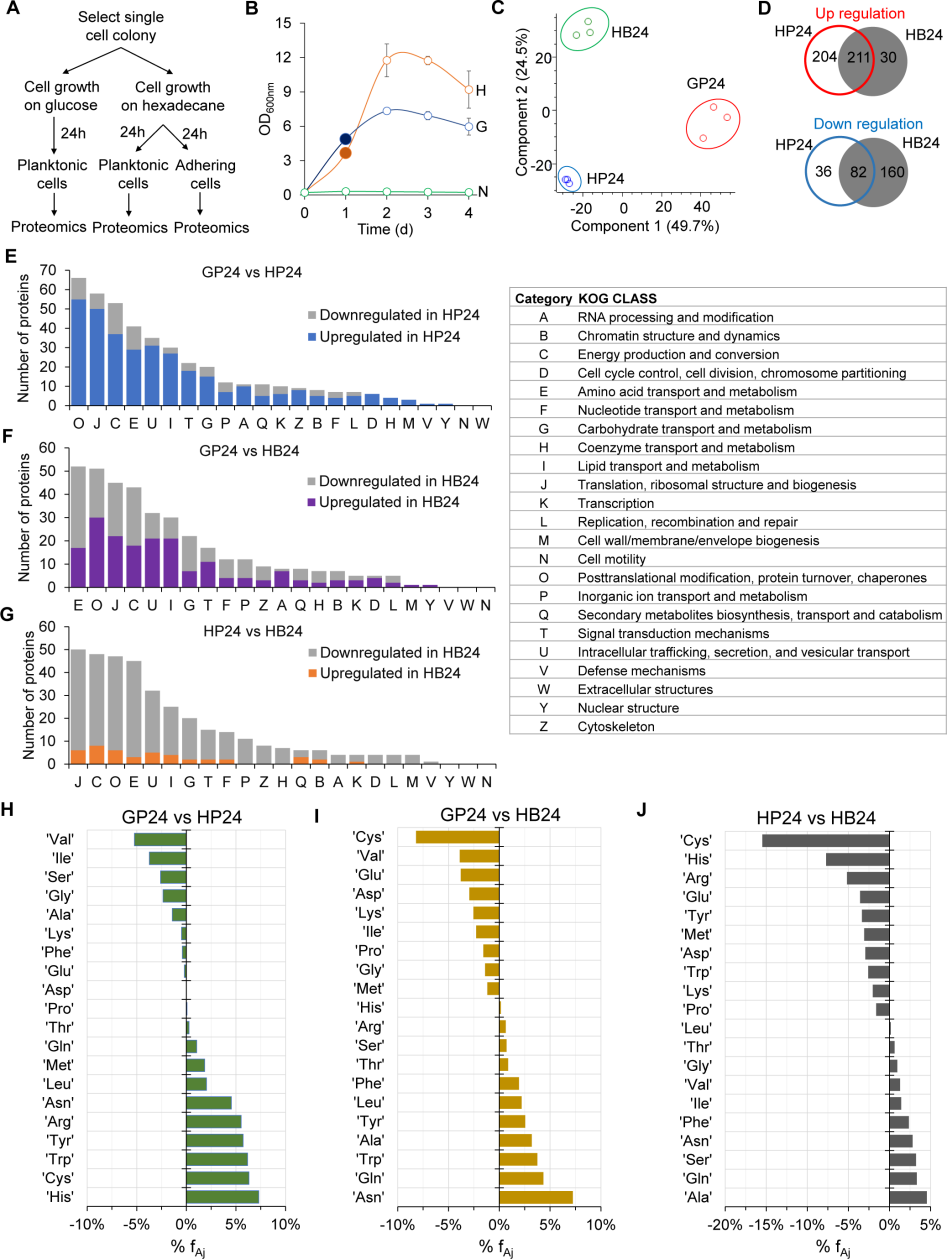
Distinct proteomes of *Y. lipolytica* cells growing on glucose and n-hexadecane. (**A**) Experimental design. (**B**) Growth and cell sample collection for proteomic analysis. Filled circle at day 1 indicates that samples were collected for proteomic analysis. Abbreviations: H, hexadecane; G, glucose; N, no carbon. (**C**) Principal component analysis of proteomes of GP24, HP24, and HB24 cells. (**D**) Proteome comparison between GP24, HP24, and HB24 cells. Regulated proteins between two biological conditions are defined with *P*-values less than 0.05 and log_2_ fold changes that are either greater than 1 (upregulated proteins) or less than −1 (downregulated proteins). (**E–G**) Number of regulated proteins between (**E**) HP24 and GP24, (**F**) HB24 and GP24, and (**G**) HB24 and HP24. (**H–J**) Percent change of mass fraction of amino acids in the measured proteomes between (**H**) HP24 and GP24, (**I**) HB24 and GP24, and (**J**) HP24 and HB24.

To elucidate the metabolic capability of the adapted *Y. lipolytica* for oil upcycling, we first compared proteomes of (i) planktonic cells (GP24) growing on glucose (control), (ii) planktonic cells (HP24) growing on n-hexadecane, and (iii) bound (or oil-adhering) cells (HB24) growing on n-hexadecane during the exponential growth phase after day 1 (Fig. 4A and B). Principal component analysis (PCA) showed that GP24, HP24, and HB24 cells exhibited distinct proteomes (Fig. 4C). Both HP24 and HB24 cells shared 211 commonly upregulated and 82 commonly downregulated proteins as compared with GP24 cells (Fig. 4D). The annotated, differentially expressed proteins represented all of the 23 Eukaryotic Orthologous Group (KOG) classes but had distinct up-/downregulation patterns based on the growth conditions (Fig. 4E, F and G). The HP24 cells had 415 upregulated proteins (log_2_ > 1, *P* < 0.05, Student’s *t*-test) and 118 downregulated proteins (log_2_ < −1, *P*-value < 0.05, Student’s *t*-test) belonging to numerous KOG classes (Fig. 4D and 2A; Data Set S1). However, HB24 cells upregulated 241 proteins and downregulated 242 proteins (Fig. 4F and 2B; Data Set S2). Pairwise comparison between planktonic HP24 and bound HB24 cells showed that HP24 cells had 388 upregulated proteins and 71 downregulated proteins (Fig. 4G and 2C; Data Set S3). Hierarchical clustering across all of the samples also confirmed distinct differences between the proteomes of planktonic and bound cells (Fig. S2D).

#### Investment of amino acids in proteomes of adapted *Y. lipolytica* varied during growth across different environmental conditions

Analyzing mass fractions of amino acids measured across each sample-specific proteome further demonstrates the global reprogramming of cellular metabolism of adapted *Y. lipolytica* growing in different environmental conditions. Comparisons included glucose versus hexadecane growth and planktonic versus oil-bound growth. As compared with the GP24 cells, the mass fraction of amino acids in the measured proteome of HP24 cells increased by 5% or more for Arg (+5.53%), Tyr (+5.72%), Trp (+6.17%), Cys (+6.31%), and His (+7.30%) and decreased 5% or more for Val (−5.23%) (Fig. 4H). In contrast, the mass fraction of amino acids in the measured proteome of HB24 cells decreased by 5% or more only for Cys (−8.20%) while it increased 5% or more for Asn (+7.23%) relative to GP24 (Fig. 4I). When comparing the planktonic (HP24) and bound (HB24) cells growing on oil, the mass fraction of amino acids in the measured proteome in HB24 cells decreased by 5% or more for Arg (−5.17%), His (−7.72%), and Cys (−15.49%) (Fig. 4J). The large percentage changes in specific amino acids indicate an alteration in overall cellular resource utilization when *Y. lipolytica* reallocated its proteome to compensate for different environmental conditions. Modulating the availability of these more heavily utilized amino acids via either targeted optimization of their biosynthetic pathway(s) or external supplementation can improve the fitness of *Y. lipolytica* for growth on hydrocarbons and/production of a target biochemical.

#### Proteome reallocation of energy and lipid metabolism enabled adapted *Y. lipolytica* to grow efficiently on n-hexadecane

**Fig 5:**
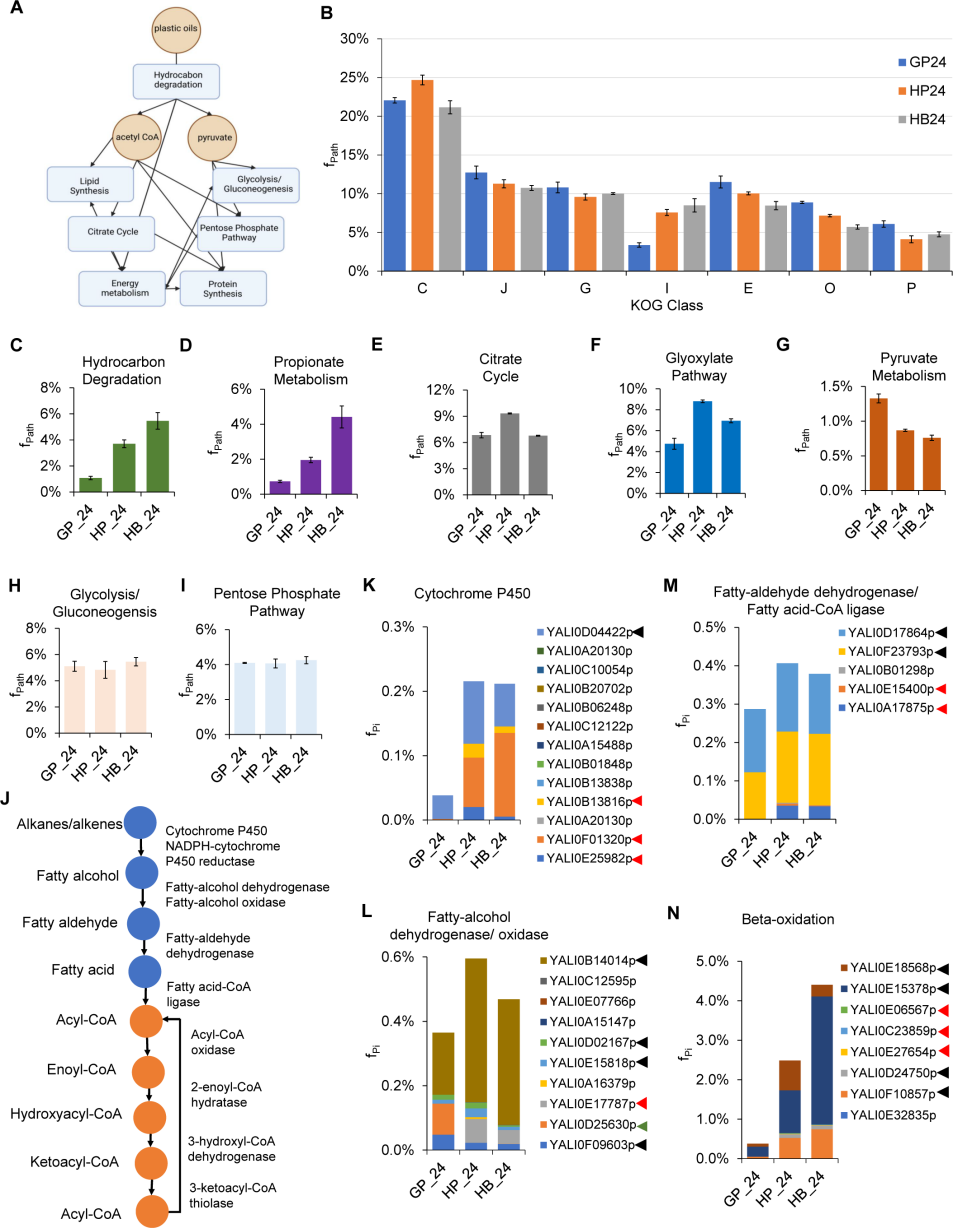
Analysis of proteome reallocation enabling robust growth of *Y. lipolytica* on hydrocarbons. (**A**) Key metabolic pathways for catabolic and anabolic metabolism of hydrocarbon utilization. (**B–**I) Comparison of mass fractions of proteomes invested in (**B**) the top seven KOG classes, (**C**) hydrocarbon degradation pathway, (**D**) propionate metabolism, (**E**) citrate cycle, (**F**) glyoxylate pathway, (**G**) pyruvate metabolism, (**H**) glycolysis/gluconeogenesis, and (**I**) pentose phosphate pathway for growth on glucose, n-hexadecane, and DP oil. (**J**) Metabolic pathway for hydrocarbon degradation. (**K–N**) Mass fractions of (**K**) cytochrome P450s, (**L**) fatty alcohol dehydrogenases/oxidases, (**M**) fatty aldehyde dehydrogenases/fatty acid-CoA ligases, and (**N**) beta-oxidation proteins in the measured proteomes. Symbols: black arrows: proteins present in GP24, HP24, and HB24; red arrows: proteins present in HP24 and HB24 only; and green arrow: proteins present in GP24 only.

To understand how GP24, HP24, and HB24 cells invested cellular resources for growth on different carbon sources, we analyzed the amino acid mass fractions of proteins classified across 23 KOG classes. Regardless of growth conditions, we found that the top seven KOG classes—including (i) Energy production and conversion (C), (ii) Translation, ribosomal structure and biogenesis (J), (iii) Carbohydrate transport and metabolism (G), (iv) Lipid transport and metabolism (I), (v) Amino acid transport and metabolism (E), (vi) Posttranslational modification, protein turnover, chaperones (O), and (vii) Inorganic ion transport and metabolism (*P*)—made up about 70%–75% of the total measured proteome abundance in adapted *Y. lipolytica* (Fig. 5B and Fig. S3). Among the KOG classes, proteomes exhibited the greatest resource allocation in energy metabolism (C) and protein biosynthesis (J, E, and O).

When growing on n-hexadecane, HP24 cells increased proteome allocation in the lipid transportation and metabolism KOG class (I) by 124% and energy production and conversion KOG class (C) by 12% relative to GP24 cells. This resource allocation came at the expense of other KOG classes (Fig. 5B). For the KOG class I, there was a total of 27 upregulated proteins (log_2_ > 1, *P* < 0.05, Student’s *t*-test) (Data Set S1) that are involved in hydrocarbon transport and degradation. For the top five upregulated proteins, YarlipO1F2_226951 (Q6C4Y2, YALI0E22781p, oxysterol-binding protein) was upregulated by 68-fold (*P* = 0), YarlipO1F2_193467 (Q6CHL1, YALI0A07733p, enoyl-CoA isomerase) by 60-fold (*P* = 0.0011), YarlipO1F2_230457 (F2Z6J3, YALI0F01320p, cytochrome P450) by 55-fold (*P* = 0.0038), YarlipO1F2_232715 (O74936, YALI0D02475p, acyl-CoA oxidase) by 48-fold (*P* = 0.0012), and YarlipO1F2_277873 (Q6CGL4, YALI0A18337p, lysophospholipase) by 37-fold (*P* = 0).

For the KOG class C, 37 proteins were upregulated (log_2_ > 1, *P* < 0.05, Student’s *t*-test) (Data Set S1); these highly upregulated proteins belong to the electron transport and Krebs cycle. Among these, the top five upregulated proteins include the following: YarlipO1F2_232437 (Q6C877, YALI0D22022p, mitochondrial F1F0-ATP synthase) upregulated by 376-fold (*P* = 0.0041), YarlipO1F2_208678 (Q6C4G4, YALI0E27005p, pyruvate dehydrogenase) by 60-fold (*P* = 0.0183), YarlipO1F2_233609 (Q6CCU5, YALI0C06446p, proteins containing the FAD binding domain) by 55-fold (*P* = 0.0129), YarlipO1F2_238445 (Q6CGH0, YALI0A19448p, aldehyde dehydrogenase) by 48-fold (*P* = 0.0044), and YarlipO1F2_86039 (Q6C7X2, YALI0D24629p, and acyl carrier protein by 38-fold (*P* = 0.0057). For HB24 cells, only lipid transportation and metabolism (KOG class I) increased up to 152% relative to GP24 cells (Fig. 5B). There was a total of 21 upregulated proteins; like HP24 cells, the highly upregulated proteins of HB24 cells participated in the hydrocarbon transport and degradation (Data Set S2).

For the pairwise comparison between the planktonic (HP24) and bound (HB24) cells growing on n-hexadecane, we found that HB24 cells increased their proteome allocation in three KOG classes: 5% in the carbohydrate transport and metabolism (KOG class G), 12% in the lipid transport and metabolism (KOG class I), and 15% in the inorganic ion transport and metabolism (KOG class P) (Fig. 5B). Since HB24 cells aggregated upon and sequestered n-hexadecane directly, higher proteome investments were made into these KOG classes, which are likely beneficial for growth on hydrophobic substrates.

#### Hexadecane induced a proteome reallocation toward hydrocarbon degradation, Krebs cycle, glyoxylate cycle, and propionate metabolism

To support cell growth and maintenance, living cells are required to synthesize the key precursor metabolites, such as acetyl CoA and pyruvate, which are important to make cellular building blocks (Fig. 5A). Further examination of the perturbed KOG classes using annotated KEGG pathways/metabolism provides a more granular view of how *Y. lipolytica* reprogrammed their metabolism to utilize n-hexadecane. To grow on n-hexadecane, *Y. lipolytica* reallocated a significant mass fraction of its proteome into the hydrocarbon degradation pathway (Fig. 5C). Relative to HP24, HB24 cells invested their proteome resources toward hydrocarbon degradation by 1.5-fold and compared with GP24 by 5.1-fold. This result showed that not only was a greater proteomic investment made in hydrocarbon degradation but also that more proteins were present and upregulated.

The metabolic pathway for hydrocarbon degradation is shown in Fig. 5J. *Y. lipolytica* has a set of 12 cytochrome P450s that enable it to grow on a wide range of paraffins by oxidizing them into fatty alcohols (57, 58). We found that *Y. lipolytica* utilized a subset of four proteins including YALI0E25982p, YALI0F01320p, YALI0B13816p, and YALI0D04422 where the first three proteins were unique for growth on n-hexadecane (Fig. 5K). Among the set of nine fatty alcohol dehydrogenases and one alcohol oxidase used to synthesize fatty aldehydes, four of these proteins including YALI0F09603p, YALI0E15818p, YALI0D02167p, and YALI0B14014p were utilized for growth on both glucose and n-hexadecane and YALI0D25630p was unique to growth on glucose while YALI0E17787p was unique to growth on n-hexadecane (Fig. 5L). To convert fatty aldehydes to fatty acids, *Y. lipolytica* utilized three (YALI0A17875p, YALI0E15400p, and YALI0F23793p) out of four available proteins where YALI0A17875p and YALI0E15400p were unique to growth on n-hexadecane, and YALI0F23793p was the only protein expressed when grown on glucose (Fig. 5M). Also included in Fig. 5M was the only annotated fatty acid-CoA ligase (YALI0D17864p) that would convert fatty acids to fatty acyl-CoA. This protein is present whether grown on glucose or n-hexadecane (Fig. 5M). *Y. lipolytica* utilized seven out of eight proteins in the beta-oxidation pathway where three of these proteins, namely, YALI0E27654p, YALI0C23859p, and YALI0E06567p, were unique to growth on n-hexadecane (Fig. 5N).

In exploring the other KEGG pathways, we observed a similar trend for propionate (C3) metabolism (Fig. 5D and Fig. S4), which is unexpected for growth on n-hexadecane that contains an even and saturated carbon chain. For the Krebs or citrate cycle, which is important for energy generation, only HP24 cells, but not HB24 cells, allocated more proteome resources than GP24 cells by 1.4-fold (Fig. 5E). Both HP24 and HB24 cells induced a greater proteomic investment in the glyoxylate pathway than GP24 cells (Fig. 5F), which is known to be important for growth on hydrophobic substrates (e.g., hydrocarbons, lipids, and fatty acids) (56) and C2 metabolism (i.e., acetate) (62). In contrast, pyruvate metabolism, which is responsible for converting pyruvate to acetyl CoA, exhibited reduced proteomic resource demand when cells grew on n-hexadecane (Fig. 5G). The combined upregulation of the Krebs cycle and downregulation of pyruvate metabolism helps explain the observed high citrate production when cells grew on n-hexadecane (Fig. 3B). Proteome allocation for glycolysis/gluconeogenesis and pentose phosphate pathway remained similar regardless of cells growing on either hexadecane or glucose (Fig. 5H and I).

#### Growth inhibition of *Y. lipolytica* by DP oil can be explained by proteome reallocation

**Fig 6:**
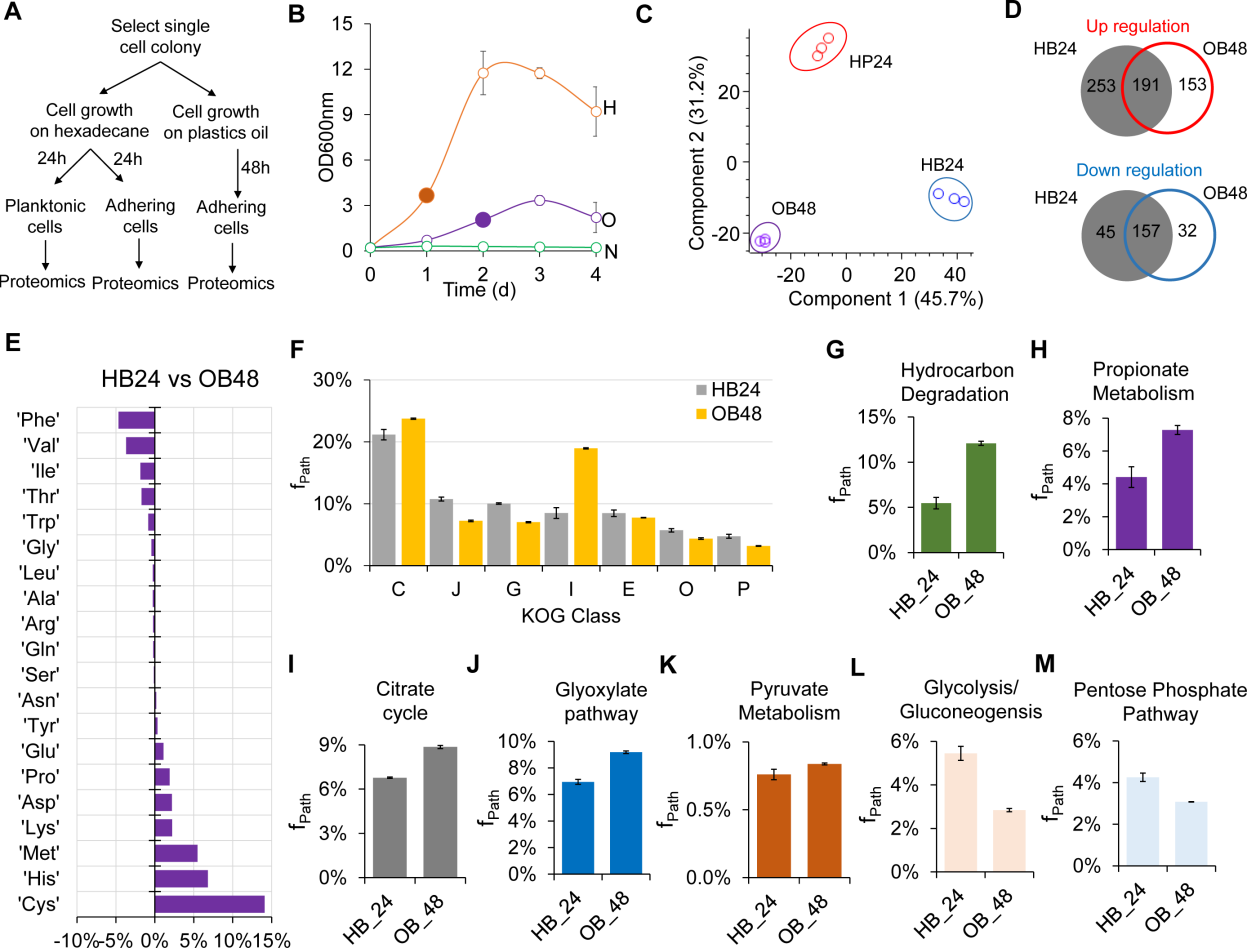
Imbalanced proteome reallocation inhibiting growth of *Y. lipolytica* on DP oil. (**A**) Experimental design. (**B**) Growth and cell sample collection for proteomic analysis. The filled circle indicates that samples were collected for proteomic analysis. Abbreviations: H, hexadecane; O, DP oil; N, no carbon. (**C**) Principal component analysis of proteomes of HP24, HB24, and OB24 cells. (**D**) Proteome comparison between HP24, HB24, and OB48 cells. Regulated proteins between two biological conditions are defined with *P*-values less than 0.05 and log_2_ fold changes that are either greater than 1 (upregulated proteins) or less than −1 (downregulated proteins). (**E**) Percent change of mass fraction of amino acids in the measured proteomes for comparison between HB24 and OB48. (**F–**M) Comparison of mass fractions of proteomes invested in (**F**) the top seven KOG classes, (**G**) hydrocarbon degradation pathway, (**H**) propionate metabolism, (**I**) citrate cycle, (**J**) glyoxylate pathway, (**K**) pyruvate metabolism, (**L**) glycolysis/gluconeogenesis, and (**M**) pentose phosphate pathway for growth on n-hexadecane and DP oil.

To better understand the inhibitory effect of DP oil, we compared the proteomes of oil-bound HB24 and OB48 cells growing on n-hexadecane and DP oil, respectively (Fig. 6A–B). At a high level, we observed distinct proteomes across HB24 and OB48 cells as depicted by the PCA plot (Fig. 6C) and differential expression of a large number of proteins (Fig. 6D, Data Set S4). Pairwise comparison between HB24 and OB48 cells showed that the mass fraction of amino acids in the measured proteome of OB48 cells increased by 5% or more for Met (+5.48%), His (+6.80%), and Cys (+14.09%) relative to HB24 (Fig. 6E).

In contrast to growth on glucose and n-hexadecane, the energy production and conversion KOG class C (*f*_Path_ = 23.74%) and the lipid transport and metabolism KOG class I (*f*_Path_ = 18.95%) exhibited the highest proteome allocations among the top seven KOG classes (C, J, G, I, E, O, and P) that represent 70%–75% of the total measured proteomes (Fig. 6F). For growth on DP oil that contains a complex mixture of hydrocarbons, we observed a significant proteome reallocation toward KOG classes C and I by 12% and 123%, respectively, at the expense of other KOG classes (J, G, E, O, and P) that are important for growth and cellular biosynthesis. For instance, translation, ribosomal structure and biogenesis (KOG class J), posttranslational modification, protein turnover, chaperones (KOG class O), and amino acid transport and metabolism (KOG class E) showed decreases in proteome allocation by 33%, 24%, and 8%, respectively. We also observed that KOG classes G (carbohydrate transport and metabolism) and P (inorganic ion transport and metabolism) were significantly reduced by 30% and 33%, respectively. Taken together, the rebalanced resource investment imparted by an over-investment in KOG classes C and I might have contributed to the growth inhibition observed when *Y. lipolytica* grew on DP oil (Fig. 6B).

Further examination of the metabolism of the KOG classes C and I show that OB48 cells exhibited significant proteome reallocation toward both the hydrocarbon degradation pathway and propionate metabolism by 2.21-fold (*P* = 0, Student’s *t*-test) and 1.65-fold (*P* = 0.001) respectively, as compared with the HB24 cells (Fig. 6G and H). The Krebs cycle, glyoxylate shunt, and pyruvate metabolism were also increased by 1.31-fold (*P* = 0), 1.32-fold (*P* = 0), and 1.10-fold (*P* = 0.0137), respectively (Fig. 6I, J, and K). In contrast, both glycolysis/gluconeogenesis and pentose phosphate pathway were reduced by 1.92-fold (*P* = 0.0001) and 1.38 (*P* = 0.0003), respectively (Fig. 6L and M). These results further explained the negative effect of a complex mixture of DP oil on the growth of *Y. lipolytica*.

## DISCUSSION

Increased demand and production of plastic have led to a significant increase in plastic waste. The negative environmental impact of plastic waste necessitates sustainable and economical strategies to recycle it. This study aimed to elucidate and harness robust metabolic capabilities of *Y. lipolytica* for biological upcycling of polyethylene derived from plastic waste into high-value chemicals. To overcome the slow kinetics of polyethylene degradation by microorganisms, we demonstrated that hybrid chemical and biological catalysis can offer a promising route for polyolefin upcycling, especially by improving the rate of polymer deconstruction from years to days. Using SiC as a catalyst, catalytic depolymerization of polyethylene resulted in oil/wax that contains mainly saturated hydrocarbons suitable for biological conversion. By maintaining DP oil in frozen storage conditions to avoid oil degradation and adapting *Y. lipolytica* to DP oil, we demonstrated that *Y. lipolytica* could grow and produce citric acid and neutral lipids from DP oil. *Y. lipolytica* could effectively consume a wide range of saturated n-alkanes and some 1-alkenes in DP oil/wax, some of which have not yet been characterized prior to this study. By analyzing and comparing proteomes of cells growing on glucose, n-hexadecane, and DP oil, we elucidated the unique metabolic capabilities of *Y. lipolytica* for upcycling polyolefins into high-value chemicals.

One key advantage of catalytic depolymerization is control over carbon chain length and product distribution by manipulating catalyst design and operating conditions. For biological upcycling using *Y. lipolytica*, DP oil with saturated n-alkanes containing n-hexadecane is highly preferred. For the same carbon chain lengths (e.g., 1-C13 and 1-C16), *Y. lipolytica* grew better on n-alkanes than the batch of 1-alkenes tested; *Y. lipolytica* did not exhibit growth on 1-C11 and 1-C22 alkenes. As a result, biological upcycling of DP oil can benefit by an introduction of a catalytic hydrogenation step to generate saturated hydrocarbons. The bi-modal molecular distribution of hydrocarbons observed in our catalytic depolymerization (Fig. 1D) implies that mid-chain stress dynamics contributed to the catalytic cleavage (63–67). If mid-chain stress was not playing a role, there would be no selectivity for C-C cleavage beyond that of end-chain and inner-chain bonding. With the former known to be more energetically demanding, a flatter molecular distribution would be expected (68–70). This result suggests that polymer chain dynamics could play a significant role in determining the molecular distribution of hydrocarbons within the oil product and may provide an avenue for improved catalytic performance if appropriate surface chemistry(s) could be isolated. While pyrolysis and hydrothermal thermal processing have been recently explored to generate product intermediates for biological upcycling in *Y. lipolytica*, these types of pretreatment require operating conditions at higher temperatures and pressures than catalytic depolymerization and generates oxygenated compounds and cyclic molecules that are more inhibitory to microorganisms (71, 72).

To utilize hydrocarbons, *Y. lipolytica* grew as a mixture of oil-bound and planktonic cells. This phenomenon occurred once the hydrophobic layer became saturated with bound cells (51). Consistent with literature (73), we observed direct evidence via microscopic images of a surface-mediated transport mechanism enabling cells to access and assimilate hydrocarbons (Fig. 2E and F). The two cell populations exhibited very distinct proteomes (Fig. 4C). We found that proteome reallocation toward both energy and lipid metabolism was critical for robust growth of *Y. lipolytica* on DP oil with n-hexadecane as the most preferential substrate (Fig. 4–6). *Y. lipolytica* expressed and upregulated many different proteins in the upstream catabolic pathways (i.e., the hydrocarbon degradation pathway, Krebs cycle, electron transport chain, and propionate metabolism). For instance, the oxysterol-binding protein (Q6C4Y2, YarlipO1F2_226951, or YALI0E22781p) of lipid metabolism was upregulated by 68-fold, which is speculated to play a role in intracellular hydrocarbon transport across organelle membranes (74). Likewise, the mitochondrial F1F0-ATP synthase (Q6C877, YarlipO1F2_232437, or YALI0D22022p) of energy metabolism was upregulated by 376-fold, which is critical for energy generation from NADH derived from the beta-oxidation of fatty acids.

While DP oil contains rich carbon and energy sources, it is inhibitory to microorganisms. The source of microbial inhibition was caused by the complex mixture of hydrocarbons (i.e., alkanes and alkenes) in DP oil. Wild-type *Y. lipolytica* exhibited poor growth on DP oil even with glucose supplementation (Fig. 2B), indicating that the toxic components (e.g., shorter chain n-alkanes and alkenes) in DP oil might have inhibited cell growth (Fig. 2G and 1). Short-term adaptation improved cell growth on both DP oil and n-hexadecane (a model hydrocarbon substrate) (Fig. 2C and D), suggesting hydrocarbon metabolism was cryptic and required activation as previously observed in both hydrophobic (75) and hydrophilic (76) substrate metabolism. *Y. lipolytica* grew more robustly on n-hexadecane than DP oil because utilizing DP oil required expression of more specialized enzymes to assimilate the mixture of complex hydrocarbons. Proteome analysis suggested that a significant increase in resource investment of energy and lipid metabolism at the expense of protein synthesis may have resulted in the observed growth inhibition. Tuning metabolic fluxes for both the catabolic and anabolic processes together with culture condition optimization is thus critical for balancing the resource allocation and hence improving the upcycling of DP oil in future studies. One simple but effective strategy is to perform long-term adaptive laboratory evolution of *Y. lipolytica* growing on DP oil based on growth selection.

Another potential source of microbial inhibition can be caused by chemical stability of DP oil that contain unsaturated hydrocarbons. We found that the adapted *Y. lipolytica* could not grow on DP oil stored at room temperature for a long period of time (>3 months). However, DP oil was stable and did not inhibit cell growth if stored frozen immediately after catalytic depolymerization.

Albeit with room for improvement, *Y. lipolytica* showed promise as a biomanufacturing microbial platform for upcycling plastic waste into higher-value chemicals such as citric acid and neutral lipids (Fig. 3). We observed drastic differences in cell growth and hydrocarbon assimilation between cultures growing on n-hexadecane and DP oil. Cells grown with n-hexadecane consumed all of the substrate, produced ~6-fold more citric acid, and achieved ~3.5 greater cell mass as compared with cells grown on DP oil. Interestingly, while GCMS analysis revealed most of the alkanes and alkenes in DP oil were consumed, DP oil-grown cells did not achieve similar cell growth or citric acid titers as compared with n-hexadecane-grown cells. We speculate that assimilated DP oil was either converted and secreted as an extracellular emulsifier (e.g., liposan) (77), functionalized into an intermediate metabolite of alkane/alkene degradation (i.e., aldehydes, alcohols or fatty acids), or respired as carbon dioxide. Interestingly, *Y. lipolytica* did not grow on individual short-chain (i.e., C11) and long-chain (i.e., C22) 1-alkenes (Fig. 2G; Fig. S1), although these were assimilated when present in the more complex DP oil. Therefore, the question arises about why *Y. lipolytica* lacks the metabolic capacity required to support cell growth on these specific alkenes. This represents a major challenge necessary to optimize cell growth and product formation from DP oil, which will be investigated in our future studies.

In summary, our work reveals that *Y. lipolytica* has metabolic capabilities that position it as a biomanufacturing platform for upcycling plastic waste-derived DP oil into higher-value chemicals. Our presented findings will help optimize catalytic depolymerization parameters to target medium-chain hydrocarbons while avoiding the generation of potentially unstable and/or inhibitory components that impact efficient biological upcycling. Future work focusing on the specific mechanisms and genes governing accessibility, uptake, and regulation of hydrocarbon metabolism is critical to unlock the full potential of *Y. lipolytica* for upcycling plastic waste (78). Rewiring robust metabolism of *Y. lipolytica* to achieve balanced resource investment for DP oil upcycling is necessary to overcome growth inhibition through medium optimization, rational strain engineering and/or adaptive laboratory evolution.

## MATERIALS AND METHODS

### Strains

The parent *Y. lipolytica* Po1f was obtained from the American Type Culture Collection (ATCC MYA-2613) and was used as the parent strain for short-term adaptation to create the adapted *Y. lipolytica* strain.

### Media and reaction conditions

#### Catalytic depolymerization of plastic waste

The catalytic cleavage of LLDPE reaction was performed under CO_2_ at atmospheric pressure to generate depolymerized plastic oil. The catalyst reaction contained LLDPE (cat# PE-NA206000, LyondellBasell, Texas, USA) and were mixed with 100 mesh unmodified SiC particles (cat# 93-1432; Strem Chemical, MA, USA) (Fig. 1B). LLDPE has an average molecular weight of 155,000 g/mol with 18 to 21 branch points per 1,000 carbon atoms where branches are comprised of methyl, ethyl, butyl, amyl, and hexyl groups. The entire reactor was vacuumed with a roughing pump for 30 minutes and then back filled with Bone Dry grade CO_2_ to atmospheric pressure three times. The catalyst bed was then heated to 400°C in 30 minutes and held for 8 hours with the condenser side kept at −77°C during the course of the reaction to promote condensation flow of the products. Following the reaction, the oil and wax were separated and purged with N_2_ and stored at −20°C. In the case of catalyst added reactions, the grease was extracted from SiC using a hot toluene bath.

#### Media for cell culturing

Defined media contained yeast nitrogen base without amino acids (Sigma #Y0626, USA) and supplemented with 380 mg/L leucine and 76 mg/L uracil. Nitrogen-limited media contained yeast nitrogen base without amino acids and ammonium sulfate (Sigma #Y1251, USA) and supplemented with 380 mg/L leucine, 76 mg/L uracil, 100 mM HEPES (Fisher #BP310, USA), 90 mM dibasic phosphate, 10 mM monobasic phosphate, and 0.73 g/L ammonium sulfate and adjusted to pH of 5 with 6 N hydrochloric acid. Glucose, DP oil, and individual hydrocarbons (Fisher Scientific, USA) were used as carbon sources at concentrations mentioned throughout the text.

#### Cell culturing conditions

All cell culturing experiments were conducted using a MaxQ6000 air incubator set to 250 rpm and 28℃. Seed cultures were generated by inoculating a single colony from a fresh petri dish in 3 mL of defined media of a 14-mL culture tube containing 20 g/L glucose and incubated overnight. Sub-seed cultures were generated by transferring 1.5 mL of the seed cultures into 50 mL of defined media of a 500-mL baffled flask containing 20 g/L glucose and incubated overnight until the mid-exponential growth phase. For nitrogen-limited experiments, sub-seed cultures were incubated for 2 days in nitrogen-limited media containing 20 g/L glucose. Cells were centrifuged and washed once in sterile water prior to starting main experiments in 50-mL baffled flasks with a 10-mL working volume and three technical replicates. For individual hydrocarbon experiments, 6 mM of each hydrocarbon was added to defined media.

#### Short-term adaptation of *Y. lipolytica* in DP oil

*Y. lipolytica* Po1f was first cultured in defined media with 2% (vol/vol) DP oil until growth plateaued. Cells were centrifuged at 3,500 rpm for 3 minutes and resuspended at 0.25 OD_600nm_ in a new flask containing freshly defined media with 2% (vol/vol) DP oil. Cells were incubated until achieving an OD greater than 1, and the top-performing replicate was transferred into a new flask containing defined media with 2% (vol/vol) DP oil. This transfer was repeated for a total of five times to generate a DP oil-adapted *Y. lipolytica* strain which was used for all succeeding experiments.

### Analytical methods

#### Cell growth

All cell growth results were quantified by measuring optical density at 600 nm absorbance of 250 µL cell culture in a 96-well plate using a BioTek Synergy microplate reader.

#### Lipid quantification

A 1.2-mL volume of cell cultures was transferred into a pre-weighted 1.5-mL centrifuge tube, washed twice with water, and resuspended to a total volume of 1.2 mL in water. One hundred microliter aliquots of each sample were transferred into a single well in duplicate using a 96-well plate for lipid quantification. The remaining 1 mL of the cell culture sample was centrifuged at maximum speed (13,000 × *g*) for 5 minutes before removing the supernatant to determine dry cell weight (DCW, g/L). The cell pellet was dried at 50℃ until a constant weight was obtained (e.g., 2–3 days) and used to calculate dry cell weight (DCW). To determine lipid content, a corn oil standard was prepared at a 5-mg/mL concentration in ethanol and used to generate working standards ranging 0.05–1 mg/mL in water. Two microliters of 1-µg/mL BODIPY 435/503 (Fisher #D3922, USA) in DMSO was added to each well of a microplate containing either cells or oil standard, and the plate was shaken in the dark for 15 minutes before measuring fluorescence (excitation 485 nm; emission 528 nm). Percent of lipid accumulated inside the cell (% lipid by weight) was calculated by dividing the lipid concentration (g/L) by DCW (g/L).

#### High-performance liquid chromatography

Metabolites (i.e., mono sugars and carboxylic acids) were quantified by high-performance liquid chromatography (HPLC). One milliliter of cell culture samples were centrifuged at 13,000 × *g* for 2 minutes, and the supernatant was transferred to a new centrifuge tube. Glucose and organic acids were measured after filtering supernatant samples with 0.2-µm filters using a Shimadzu HPLC system equipped with UV and RID detectors (Shimadzu Scientific Instruments, MD, USA) and an Aminex 87H column (Bio-Rad, CA, USA) at a flow rate of 0.6 mL/minutes and oven temperature of 48℃ with 10 mN sulfuric acid mobile phase (79).

#### Gas chromatography coupled with mass spectrometer

Hydrocarbons were quantified by gas chromatography coupled with mass spectrometer (GCMS). Ten milliliters of sacrificial flask replicates was transferred into a 15-mL tube and stored at –20 ℃. Samples were thawed to room temperature before adding 2 mL of solvent (i.e., chloroform containing 100 mg/L ethyl pentadecanoate as the internal standard). Samples were vortexed and incubated at room temperature for 2 hours prior to centrifuging at 3,500 rpm for 5 minutes, extracting the organic phase, and filtering through a 0.2-µm filter. For samples cultured with n-hexadecane as the sole carbon source, 10 µL of extract was transferred into a GC vial containing 1,990 µL of solvent. For samples cultured with DP oil, 125 µL of extract was transferred into a GC vial with an insert containing 125 µL of solvent. The GC (HP 7820A, Agilent, CA, USA) equipped with a MS (HP 5977B, Agilent, CA, USA) method was used to detect n-hexadecane and DP substrates as follows: 1 µL of each sample was injected into the GC capillary column (HP-5MS, 30 m × 0.25 mm × 0.25 µm, Agilent, CA, USA) using the spitless mode at an injection temperature of 300°C. The carrier gas, helium, was set at a flow rate of 14.7 mL/minutes. The oven was set to an initial temperature of 80°C and held for 5 minutes and ramped by 30 °C/minutes to 100°C and held for 12 minutes and ramped by 40 °C/minutes to 160°C and held for 15 minutes and ramped by 40 °C/minutes to 300°C and held for 22 minutes and baked at 325°C for 10 minutes. Peak areas of extracted ions at specific retention times were used to quantify substrates as illustrated in Data Set S1. These peak areas were normalized for each individual sample by dividing by the peak area of the respective internal standard.

#### Liquid chromatography tandem mass spectrometer-based proteomic measurements

*Y. lipolytica* was grown on glucose, hexadecane, or DP oil as described above and sampled at 24 and 48 hours. Cultures were centrifuged at 4,700 × *g* for 10 minutes to isolate distinct populations of cells via phase separation (Fig. 2E). Oil-bound cells and culture supernatants were removed from the pelleted planktonic cells and adherent cells isolated from supernatants via filtration. Planktonic cells and bound cells were then processed for liquid chromatography-tandem mass spectrometry (LC-MS/MS)-based shotgun proteomics as previously detailed (49, 80). Briefly, cells were lysed via bead beating (0.5 mm zirconium oxide beads) in 100 mM Tris-HCl, pH 8.0 (Geno/Grinder 2010; SPEX). Crude lysates were then mixed with 4% SDS and 10 mM dithiothreitol and then heated to 90°C for 10 minutes. Crude lysates were then pre-cleared via centrifugation at 21,000 × *g* for 10 minutes, moved to a new microfuge tube, adjusted to 30 mM iodoacetamide, and incubated for 20 minutes at room temperature in the dark. Proteins were isolated to magnetic beads (1 micron SeraMag beads; GE Healthcare) by the protein aggregation capture (PAC) method (81), washed with acetonitrile and ethanol, and digested *in situ* with sequencing-grade trypsin [1:75 (wt/wt) trypsin to protein ratio] in 100 mM ammonium bicarbonate, pH 8.0 at 37°C overnight. Samples were digested again the following day for 4 hours. The tryptic digests were then acidified to 0.5% formic acid, filtered through a 10-kDa MWCO spin filter (Vivaspin500; Sartorius), and quantified via NanoDrop OneC at A205 nm.

Peptide samples were analyzed by automated 1D LC-MS/MS analysis using a Vanquish UHPLC plumbed directly to a nanoelectrospray source installed on a Q Exactive Plus mass spectrometer (Thermo Scientific). Peptide samples were de-salted and separated using a trapping column coupled to an in-house pulled nanospray emitter, as previously described (80). The trapping column (100 µm ID) was packed with 6 cm of 5 µm Kinetex C18 reversed phase (RP) resin (Phenomenex), while the nanospray emitter (75 µm ID) was packed with 15 cm of 1.7 mm Kinetex C18 RP resin. For each sample, 3 µg of peptides was loaded, de-salted, and separated by UHPLC along a 180-minute organic gradient (49). Eluting peptides were measured and sequenced by data-dependent acquisition on the Q Exactive MS as previously described (82).

#### Nuclear magnetic resonance

NMR experiments were performed at room temperature on a Varian VNMRS 500 MHz spectrometer. Processing of the spectra was performed using the software MNova. For sample preparation, 200 µL of the DP oil was diluted in 400 µL of deuterated chloroform (Sigma-Aldrich 99.8%).

#### Gel permeation chromatography

Gel permeation chromatography (GPC) was used to characterize the molecular weight distribution for the grease samples. The tests were carried out using a Viscotek 350A HT-GPC equipped with RI detection and using 1,2,4-trichlorobenzene at 145°C as the mobile phase.

#### Confocal imaging

Oil-bound cell samples were stained with calcofluor white and nile red. Mixing was carried out gently with a pipette tip as to minimize disruption of the 3D cell/oil matrix. After allowing to stain for 5–10 minutes, 10–20 μL of the sample was placed on a microscope cover slip which was then placed on the confocal microscope (Leica SP8 White Light Laser Confocal System) for imaging.

### Bioinformatics and computational analysis

#### Proteomic analysis

MS/MS spectra were searched against the *Y. lipolytica* pO1F v2 proteome (JGI) (83) appended with common protein contaminants using the SEQUEST HT algorithm in Proteome Discoverer v.2.3 (Thermo Scientific). Peptide spectrum matches (PSMs) were required to be fully tryptic, allowing for up to two miscleavages: a static modification of 57.0214 Da on cysteine (carbamidomethylation) and a dynamic modification of 15.9949 Da on methionine (oxidation) residues. PSMs were scored and filtered using Percolator with false-discovery rates (FDR) initially controlled at <1% at the PSM and peptide levels. Peptides were then quantified by chromatographic area under the curve, mapped to their respective proteins, and areas summed to estimate protein-level abundance. For proteome reallocation analysis, these protein abundances were used directly for the calculation without filtering and imputation. For differential expression analysis, proteins that do not have at least 70% valid values in each group were removed and the remaining proteins were log_2_ transformed using Perseus v2.0.6.0 (84). Missing values were imputed to estimate the mass spectrometer’s limit of detection in Perseus. Statistical tests (*t*-test and ANOVA) were performed with default parameter settings in Perseus. Significant differences in protein abundance were calculated for relevant sample group comparisons after FDR correction.

Though the Po1f strain and its associated proteome database were interrogated in this study, more detailed functional information is available for the *Y. lipolytica* CLIB122 reference proteome. To improve functional annotations and to view the results in the same context as the CLIB122 reference strain, the Po1f strain was analyzed with OmicsBox v2.2.4 software. This enhanced search provided BLASTP, InterPro, Gene Ontology (GO) terms, Enzyme Commission (EC) codes, Kyoto Encyclopedia of Genes and Genomes (KEGG), KOG, and Signal P information and enabled the generation of an orthology lookup table linking Po1f proteins to their orthologs in the CLIB122 reference proteome.

#### Proteome reallocation analysis

KEGG and UniProt were used to obtain protein annotation, protein sequences, and pathway ontology (85, 86, 87). The hydrocarbon degradation pathway that oxidizes hydrocarbons to fatty acids in endoplasmic reticulum and then acetyl CoA in peroxisome were manually curated based on literature sources (56, 57, 59–61).

Mass fraction of a protein *P*_*i*_, *f*_*Pi*_, in the proteome is calculated as follows (88):


(1) $$f_{Pi}=\frac{{PA}_{i}}{\sum_{i=1}^{N}{PA}_{i}}$$

where PA_*i*_ is the abundance of protein *i*, *N* is the total number of proteins in the measured proteome, and $\sum_{i=1}^{N}f_{Pi}=1$. The mass fraction of a pathway proteome, *f*_Path_, that contains M proteins is determined as follows (88):


(2) $$f_{Path}=\sum_{i=1}^{M}f_{Pi}$$

Mass fraction of amino acid *j*, *f*_*Aj*_, in the proteome is computed as follows (88):


(3) $$f_{Aj}=\frac{\sum_{i=1}^{N}{AA}_{ij}\cdot f_{Pi}}{\sum_{j=1}^{20}\sum_{i=1}^{N}{AA}_{ij}\cdot f_{Pi}}$$

where AA_*ij*_ is the abundance of amino acid *j* in protein *i* of the proteome and $\sum_{j=1}^{20}f_{Aj}=1$.

## Data Availability

All raw mass spectra for quantification of proteins used in this study have been deposited in the MassIVE and ProteomeXchange data repositories under accession numbers MSV000091396 (MassIVE) and PXD040555 (ProteomeXchange), with data files available at ftp://massive.ucsd.edu/MSV000091396/.

## SUPPLEMENTAL MATERIAL

Data Set S1 msystems.00741-23-s0001.xlsx Pairwise comparison of differential protein expression between the planktonic GP24 and planktonic HP24 cells.

Data Set S2 msystems.00741-23-s0002.xlsx Pairwise comparison of differential protein expression between the planktonic GP24 and bound HB24 cells.

Data Set S3 msystems.00741-23-s0003.xlsx Pairwise comparison of differential protein expression between the planktonic HP24 and bound HB24 cells.

Data Set S4 msystems.00741-23-s0004.xlsx Pairwise comparison of differential protein expression between the bound HB24 and bound OB24 cells.

Supplemental figures msystems.00741-23-s0005.pdf Fig. S1 to S4.

ASM does not own the copyrights to Supplemental Material that may be linked to, or accessed through, an article. The authors have granted ASM a non-exclusive, world-wide license to publish the Supplemental Material files. Please contact the corresponding author directly for reuse.
