# Supplementary material for: Proteomes reveal metabolic capabilities of Yarrowia lipolytica for biological upcycling of polyethylene into high-value chemicals
Source: mSystems. 2023 Oct 26;8(6):e00741-23. doi: 10.1128/msystems.00741-23 (PMC10734471; doi:10.1128/msystems.00741-23)

Supplementary Materials

Proteomes Reveal Metabolic Capabilities of *Yarrowia lipolytica* for Biological Upcycling of Polyethylene into High-Value Chemicals

Caleb Walker[1], Max Mortensen[1], Bindica Poudel[1], Christopher Cotter[1], Ryan Myers[1], Ikenna O. Okekeogbu[2], Richard Giannone[2], Seunghyun Ryu[1], Bamin Khomami[1], Siris Laursen[1], and Cong T. Trinh*[,1]

[1]Department of Chemical and Biomolecular Engineering, University of Tennessee, TN 37996

[2]Biosciences Division, Oak Ridge National Laboratory, Oak Ridge, TN 37831

*Corresponding author: Cong T. Trinh. Email: ctrinh@utk.edu

Figure S1: Growth kinetics of adapted *Y. lipolytica* on (**A-P**) n-alkanes and (**Q-T**) 1-alkenes. The growth experiments were performed with at least 6 biological replicates. Each value on a graph is represented by an average ± 1 standard deviation (n = 6 or 12).

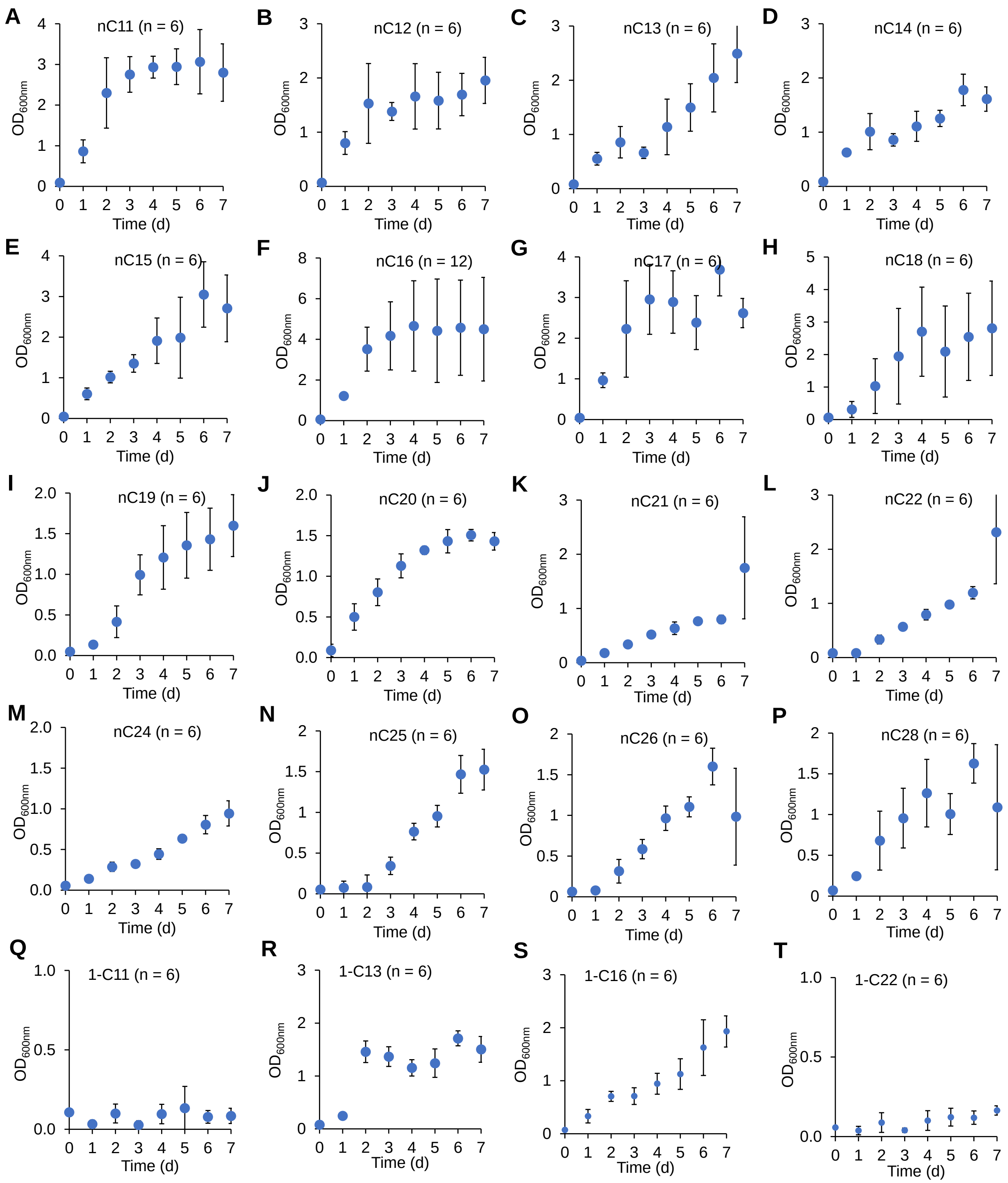

Figure S2: Comparison of proteomes of *Y. lipolytica* growing on glucose and n-hexadecane. **(A-C)** Volcano plots depicting differential protein expression between **(A)** GP24 and HP24 cells, **(B)** GP24 and HB24 cells, and **(C)** HP24 and HB24 cells. **(D)** Heatmap showing 5 distinct clusters of proteins that were differentially expressed for growth on different substrates.

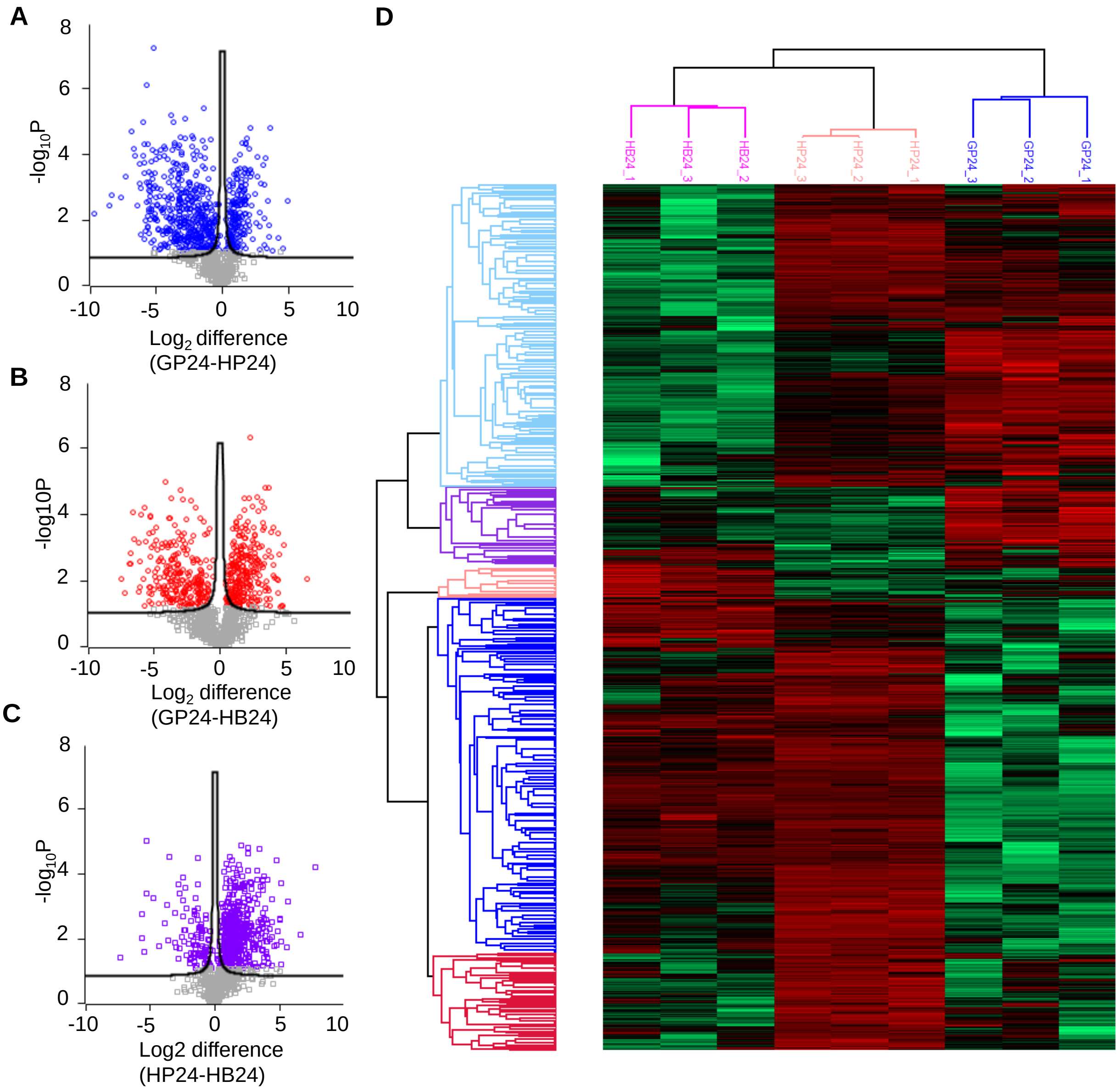

Figure S3: Comparison of mass fractions of proteomes invested in all 23 KOG classes of *Y. lipolytica* growing on glucose, n-hexadecane, and DP oil.

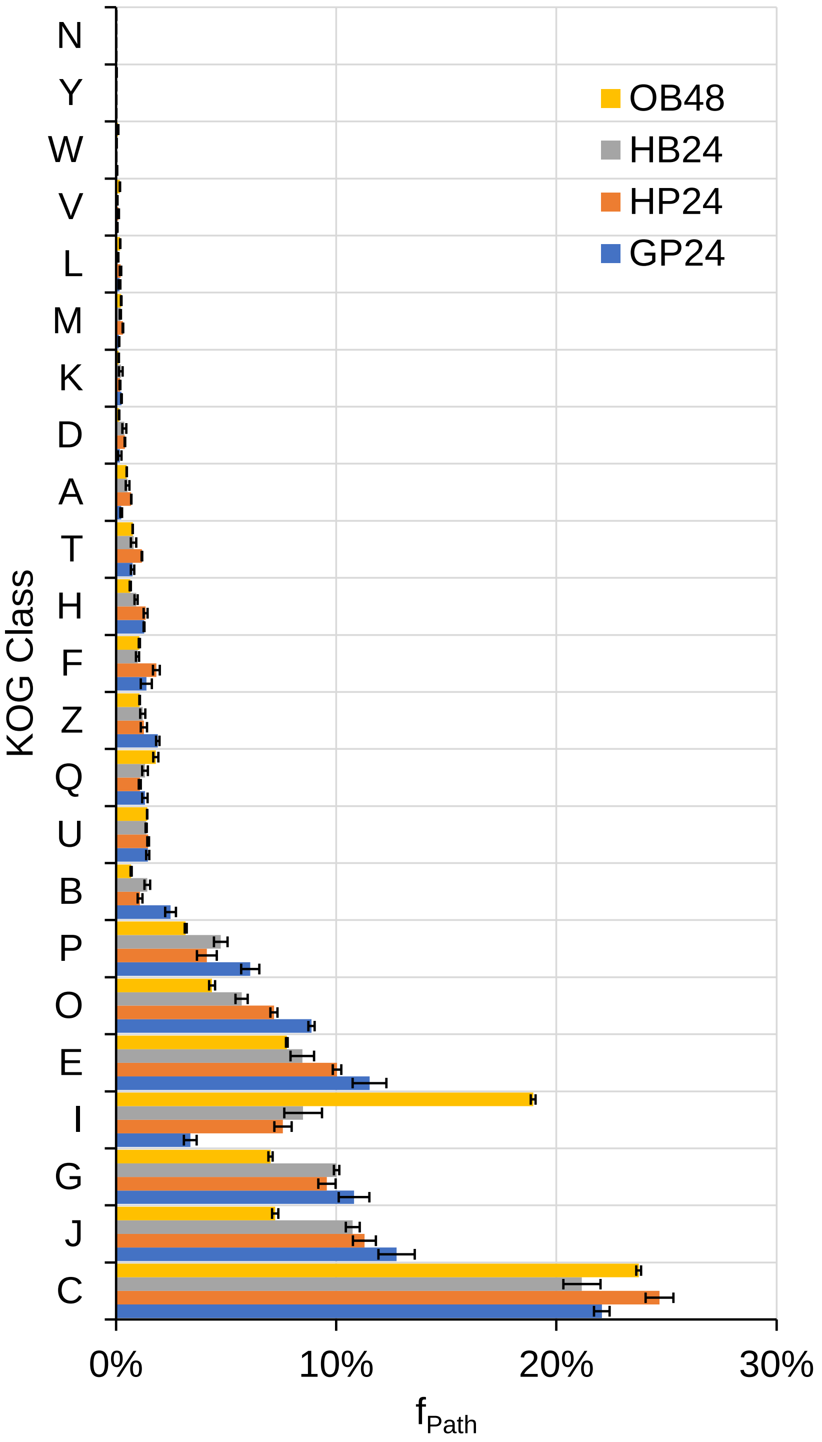

s

Figure S4: Proteome reallocation for propionate metabolism of *Y. lipolytica* growing on glucose, n-hexadecane, and DP oil. **(A)** Metabolic pathway of propionate degradation. **(B)** Mass fractions of proteins involved in the propionate metabolism.

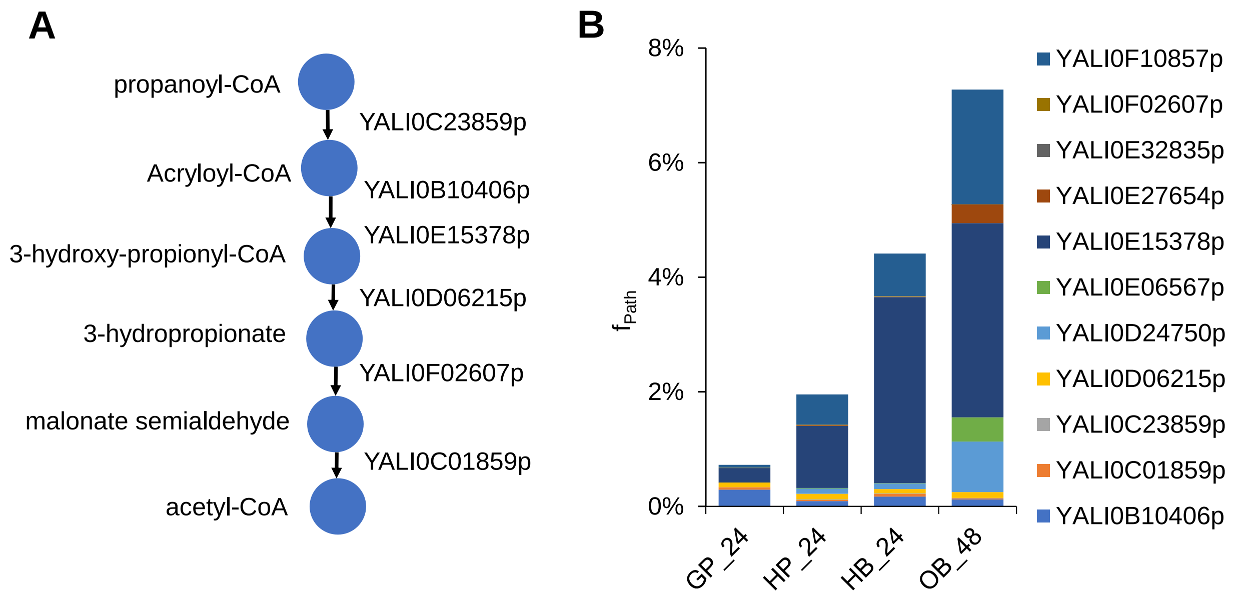

Supplement: Supplemental figures — Fig. S1 to S4. [file msystems.00741-23-s0005.pdf]
